# Going Beyond Childhood and Gender-Based Violence: Epigenetic Modifications and Inheritance

**DOI:** 10.1089/whr.2024.0010

**Published:** 2024-05-28

**Authors:** Letizia Li Piani, Edgardo Somigliana, Laila Giorgia Micci, Gaia Spinelli, Giussy Barbara

**Affiliations:** ^1^ Dept of Clinical Sciences and Community Health, Università degli Studi di Milano, Milan, Italy.; ^2^ Infertility Unit, Fondazione IRCCS Ca' Granda Ospedale Maggiore Policlinico, Milan, Italy.; ^3^ SVSeD - Service for Sexual and Domestic Violence, Fondazione IRCCS Ca' Granda Ospedale Maggiore Policlinico, Milan, Italy.

**Keywords:** childhood violence, epigenetics, epigenetic clocks, gender-based violence, sexual assault

## Abstract

Being exposed to childhood or gender-based violence is associated with subsequent adverse events in individual lives. Not only can it cause psychological distress but violence survivors suffer from a range of long-term adverse health outcomes, including higher morbidity, higher mortality, and higher risk of chronic diseases. Epigenetics may be involved in the determinisms of these long-term detrimental effects. A large body of evidence supports this biological mechanism to explain violence-related health impairment in the long term. However, studies specifically focusing on violence are scant and nonunivocal. Epigenetic modifications of genes involved in stress response and in the hypothalamus–pituitary–adrenal axis regulation are the most commonly and consistently reported. Promising evidence also emerged for the use of epigenetic clocks. Finally, although very limited, there is evidence supporting the notion that long-term health impairment may be transmitted from one generation to the other. Overall, despite promising, available evidence is yet incomplete. The overlap with pure psychological mechanisms of health impairment exposes the findings to confounders and hampers strong conclusions. Based on a literature search on PubMed/Embase, our narrative review aims to illustrate the evidence concerning the potential bond between epigenetics and violence, including also possible impacts on later generations. The goal is to encourage further research to help the development of a more holistic approach for such a vulnerable and often neglected population. Further research is warranted to precisely disentangle the role of epigenetics in mediating the long-term health impairment associated with childhood or gender-based violence. Advances in this area may open new avenues of treatment. Epigenetic modifications may indeed be reversible and could be an attractive therapeutic target to minimize the long-term consequences of childhood or gender-based violence.

## Introduction

Women and children are at greatest risk of all types of violence, whether sexual, psychological, gender-based, or family-based, and face the utmost barriers to protection and services.

### Definitions and background

Gender-based violence encompasses any form of violence directed against a person because of gender. Both women and men experience gender-based violence but as the majority of victims are women and girls, gender-based violence and violence against women are terms that are often used interchangeably.^1^

Sexual violence is often the most humiliating form of violence, and it is far more common in most societies than generally assumed.^1^ Almost one in three women worldwide has experienced sexual violence at least once in her life.^2^ Although sexual assault is the most common form of episodic violence against women and child, domestic violence can take many forms. Intimate partner violence (IPV), also referred to as domestic violence, describes any use of physical or sexual force, actual or threatened, in an intimate or domiciliary relationship, which may include a single act of violence, or a number of acts forming a pattern of abuse through the use of assault and controlling behavior.^1^ Even if IPV, where the perpetrator is a current or former intimate partner, is more likely to be associated with specific risk factors such as the belief in strict gender roles, heavy alcohol and drug use, or living in communities with high rates of poverty and limited educational and economic opportunities, violence against women cuts across all social classes and ethnic groups.^3^ Childhood abuse is extremely common,^4^ with 20% of women and nearly 10% of men reporting being victimized before the age of 18 years.^5^ Unfortunately, IPV and child abuse often coexist within the same families. Moreover, children may witness domestic violence against their mothers (“witnessed violence”) with severe long-term psychological adverse consequences.^6^

### Long-term adverse consequences

Being exposed to any form of violence heralds subsequent adverse events in individual lives.^1^ In fact, even a single episode can lead to poor behavioral and mental health outcomes for the victim, with post-traumatic stress disorder (PTSD) as one of the most prominent outcomes.^1,7^ Not only can it cause psychological distress but survivors of violence may suffer from a range of long-term adverse health outcomes, including higher morbidity, higher mortality, and higher risk of chronic diseases.^8^ It is noteworthy that this long-term health impairment may also be transmitted from one generation to the other.

### Potential role of epigenetics

Any life-experience, such as traumatic events, can affect health outcomes, and emerging evidence suggests that the environment can induce epigenetic changes that can persist for a long time, and some of these changes can also be passed on to succeeding generations.^9,10^ Besides, epigenetic aberrations can be translated into an “unhealthy” phenotype at least or contribute to disease onset, but luckily, they could be reversed, unlike genetic changes.^11^ From this perspective, epigenetics may be the possible common denominator for physical, psychological, and transgenerational effects of violence.^12^ In fact, there is evidence of a link between epigenetic changes and PTSD, as if epigenetics might play a role in physiological and behavioral responses to environmental stressors.^13^ Epigenetics refers to the heritable changes in gene expression or functionality without altering the DNA sequence.^14^ It includes DNA methylation, modifications of histone proteins, and small RNA-mediated gene silencing.^14^ Genome regulation and epigenetic expression could potentially be affected by either a single sudden violent event or an ongoing perturbation.^14^ Interestingly, the role of epigenetics in global biological aging, with the development of machine-learning models that automatically select the most informative DNA methylation regions to describe the potential gap between chronological age and the real biological age, has ushered in a new era of molecular research in the field of aging.^15,16^ These models, called epigenetic clocks, could also glean information on the consequences of traumatic events, describing possible global aging disruption rather than epimutation on single genes.^17^

Either as single-gene mutation or epigenetic age acceleration, epigenetics could provide a long-term picture on disease risk of the effects of any form of violence. One may even speculate that this tool could capture different effects based on the type of violence. It could also be a robust tool for predicting the severity of the consequences of violence. Besides, the heritable nature of these changes could be implicated in the intergenerational transmission of the detrimental effects of traumatic experiences to the survivors’ newborns. Moreover, and of utmost interest, epigenetic modifications may also be reversible. This potential reversibility makes epigenetics a high-priority area of research. Epigenetic effectors could be an attractive therapeutic target to help minimize the consequences of epigenetic disarrangements.^18^

### Scope of the review

**FIG. 1.**
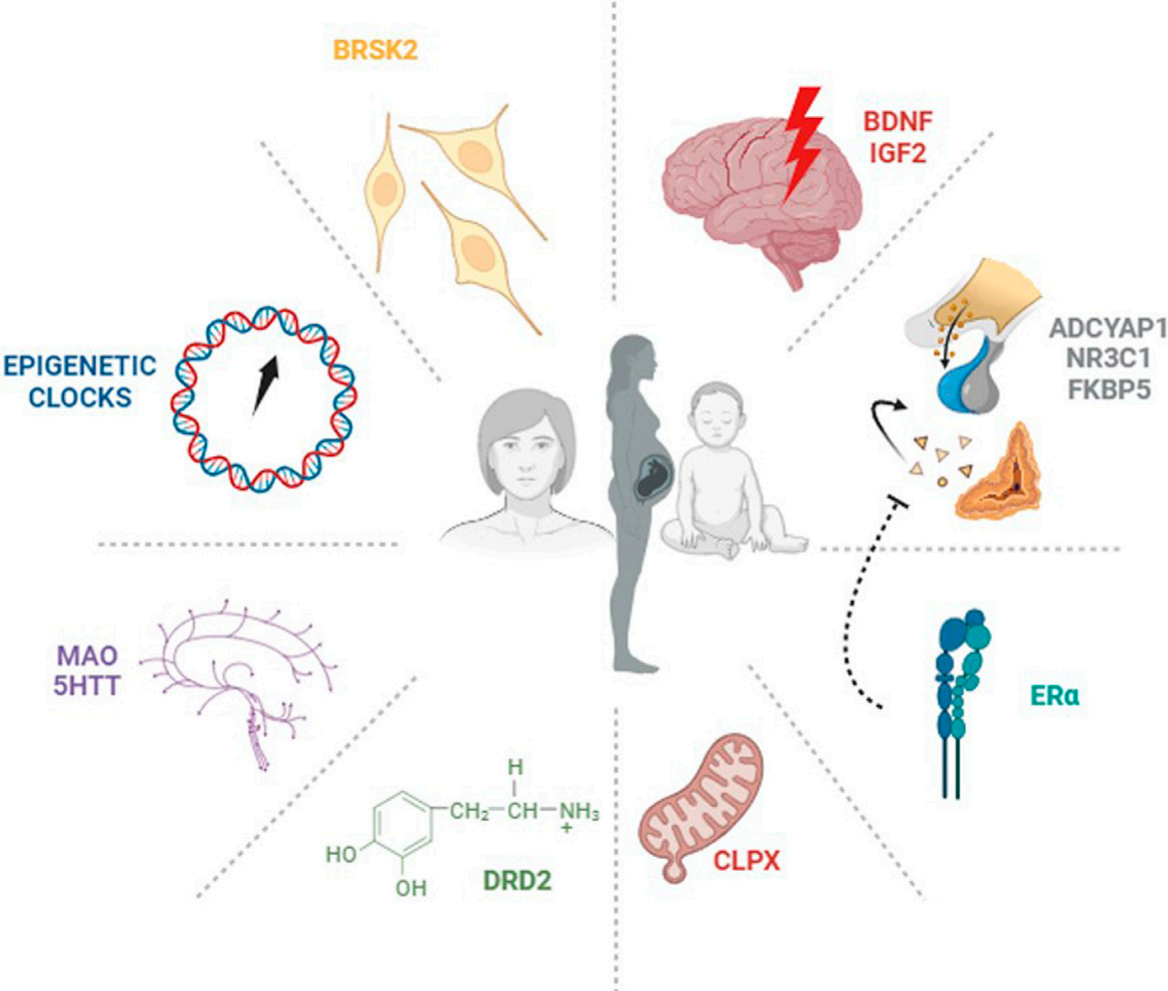
Epigenetic patterns in three potential victims (children, women, and newborns). Acronyms are depicted in Table 1.

The aim of this narrative review is to provide an overview of the evidence gathered so far on the potential bond between epigenetic patterns and violence, including gender-based IPV and violence during childhood, considering also possible impacts on later generations. The goal is to encourage further research to help in the development of a more holistic approach for such a vulnerable and often neglected population. Genes discussed in this review and their effects are listed in Table 1 and illustrated in Figure 1.

**Table 1.** Summary of the Genes Associated with Epigenetic Changes in Violence Victims

Gene	Gene definition	Function of the gene	References
BDNF	Brain-derived neurotrophic factor	Regulator of synaptic transmission and neuroplasticity. Implied in stress response, memory and learning, and in aging	Piccinini et al.,^12^ Serpeloni et al.^19^
CLPX	Caseinolytic mitochondrial matrix peptidase chaperone subunit	Regulator of mitochondrial function, implied in neuronal function. Linked with psychiatric disorder risk and aging	Serpeloni et al.^19^
BRSK2	Brain-specific serine /threonine-protein kinase 2	Neuronal development and maintenance, regulation of glucose blood levels	Nöthling et al.^20^
IGF2	Insulin-like growth factor 2	Fetal and placental growth factor, implied in memory, depression, and autism. Linked to BDNF function	Piccinini et al.^12^
ADCYAP1	Adenylate cyclase-activating polypeptide 1	Codification for PACAP, major regulator of the HPA-axis	Nöthling et al.^20^
NR3C1	Nuclear receptor subfamily 3, group C, member 1	Codification for glucocorticoid receptor, which binds cortisol, creating a negative feedback loop within the HPA-axis	Perroud et al.,^21^ Radkte et al.,^22^ Shield et al.,^23^ Marzi et al.,^24^ Serpeloni et al.,^19^ Wadji et al.,^25^ Wadji et al.^26^
ERα	Estrogen receptor	Highly expressed in hypothalamus, the pituitary, and adrenal gland, mediating negative feedback on HPA axis	Fiacco et al.^27^
FKBP5	FK binding protein 5	Encodes for heat shock protein, which is a cochaperone for glucocorticoid receptors	Misiak et al.,^28^ Marzi et al.,^24^ Konig et al.,^29^ Serpeloni et al.,^30^ Grasso et al.^31^
MAO	Monoamine oxidase A	Metabolism of serotonin and other monoamine neurotransmitters	Checknita et al.^32^
5HTT	Serotonin transporter	Reuptake of serotonin at synaptic neuron, role in PTSD and depression-susceptibility	Beach et al.^33^
DRD2	D2 dopamine receptor	Regulator of the dopaminergic signaling system, involved in reward and motivation, memory and learning	Piccinini et al.^12^
Epigenetic clocks	Horvath, Hannum, PhenoAge, GrimAge, epigenetic acceleration	Mathematical models, based on CpG methylation of different genes, able to describe biological aging that could be different from chronological one. Associated with morbidity and mortality	Lawn et al.,^34^ Hamlat et al.,^35^ Mc Crory et al.,^36^ Hamlat et al.,^37^ Shenk et al.,^38^ Quinn et al.^39^

### Selection process

The search procedure involved using the PubMed/Embase database. The following search terms were utilized across database to ensure comprehensive coverage of relevant studies on epigenetic aspects of violence, including “Epigenetic,” “Epigenetic clock,” “Sexual violence,” “Intimate partner violence,” “Child abuse,” “gender-based violence,” “Child maltreatment,” “Stress disorder,” “Victimization.” During the initial search using the specified keywords, a substantial number of articles unrelated to this literature survey were retrieved. One author (LLP) then selected the articles that were relevant. Retrospective, prospective, or cross-sectional studies were considered for this review on this topic.

## Results

### Women abuse, mental disorders, and epigenetics

Psychological problems can be associated with traumatic events such as sexual abuse. PTSD is the most common form of trauma disorder that occurs after sexual violence: it usually reaches a prevalence of between 35% and 45%, and it can persist even more than a year after the event.^2^ It can evolve into a form of lifelong anxiety associated with intrusive memories, disturbing nightmares and flashbacks, avoidance, and hyperarousal. It is probably due to an imbalance between two neurochemical systems in the brain (serotonin and substance P), and the severity depends on the degree of the gap.^40^ Given that DNA methylation patterns may affect the ability to adapt to traumatic events and consequently the risk of developing PTSD, much of the literature has focused on the molecular aspects of the disorder, thanks to epigenome-wide association studies (EWAS).^41,42^ However, precise evidence specifically focusing on women abuse is scant.

In a pilot study based on blood sample and personal questionnaire, epigenetic signatures for seven selected genes relevant to psychiatric disorders and PTSD were compared between 62 exposed and 50 nonexposed women to IPV.^12^ Three (BDNF, DRD2, and IGF2) of the seven genes considered turned out to have a methylation perturbation in the subgroup of women (23% of the cohort) who reported at least one PTSD criterion. This finding seems coherent with the role played by each gene. BDNF regulates neuroplasticity and stress-response, and a hypermethylation level has already been observed in veterans with PTSD;^40^ IGF2 likely correlates with BDNF expression, and it is implied in memory and neurofunctions as well.^41^ Similarly, DRD2 methylation dysregulation in the dopamine system has already been observed in certain PTSD presentations (*i.e.,* attention, arousal, sleep) and in case of childhood maltreatment.^42^ Although these preliminary data should be confirmed, the authors suggest that certain epigenetic signatures implied in neuro-functions and memory could be involved in response to stress in the context of sexual violence-related PTSD.

In an earlier study focused on the epigenetics of PTSD following IPV, Nöthling et al. examined a cohort of rape survivors, with similar sociodemographic background and ethnicity.^20^ They performed an EWAS analysis after 3 months from the event since it is considered the first time point useful for PTSD occurrence. Among the 34 differentially methylated regions found, they focused the attention on the following two genes: brain-specific serine/threonine-protein kinase 2 (BRSK2) and adenylate cyclase-activating polypeptide 1 (ADCYAP1), which have already been linked to PTSD. In fact, BRSK2 is associated with neuronal development and glucose regulation, likely justifying the increased risk for diabetes and cardiovascular disease in PTSD,^43^ whereas ADCYAP methylation is negatively correlated with a major regulator of the hypothalamus–pituitary–adrenal (HPA) axis and stress, and it has already been associated with PTSD severity.^44^ Although based on a small sample size (*n* = 24 PTSD raped women vs. *n* = 24 non-PTSD raped women), the authors observed hypomethylation status of both genes in those victims who developed PTSD symptoms. Indeed, the more ADCYAP1 methylation was decreased, the higher the severity of PTSD symptoms at 6 months.^20^

Similar to PTSD, other mental disorders may be related to neuronal dysfunctions after traumatic events, such as major depressive disorder (MDD), conduct disorder, and alcohol abuse.^32,45,46^ MDD is commonly associated with sexual abuse, affecting individuals who experienced either IPV or non-IPV at twice the rate of those who have not experienced any form of trauma.^45^ As already observed in mental disorders, one research group decided to study the methylation profile of the enzyme monoamine oxidase (MAO), which metabolizes serotonin.^32^ Based on 114 young Swedish women with and without sexual violence, the authors described the grade of methylation according to the type of abuse. Both sexual abuse and depressive symptoms resulted linked to hypermethylation levels of MAO. The association remained robust even after adjusting for potential confounders (psychoactive medication, alcohol, and drug abuse).^32^

Another pathway that may be central to the women response to IPV is the function of the HPA axis. While acute stress activates the pituitary–adrenal system, increasing cortisol production, chronic stress could provoke inactivity of the HPA axis, leading to a permanent deprivation of its reactivity.^47,48^ Not surprisingly, NRC31, which regulates glucocorticoid receptor depending on stress exposure, has been extensively studied. A link between IPV and cortisol trajectories has been a special focus especially in women with childhood abuse history, although with heterogeneous methodologies and nonunivocal results.^49–51^ A further step forward was taken by Wadji et al., who queried whether NRC31 methylation could be different according to symptoms. Based on saliva samples and a mental health questionnaire in a cohort of 40 mother–child dyads in Cameroon, a significant correlation between anxiety levels and methylation grade of NR3C1 promoter was reported in women exposed to IPV compared with the control group, while no changes were shown in children.^26^

Collectively, these data support a role of neurobiological mechanisms behind sexual abuse, especially in the context of IPV, and potential susceptibility to a variety of psychiatric disorders. Evidence is highly promising but not univocal, possibly because of confounders (choice of the control group, timing and modality of sample collection, type of abuse, confounders such as pretrauma psychiatric disorders or social degradation), insufficient study power, and the confounding effects of the overlapping pure psychological effects (not all psychological mechanisms may obviously have an epigenetic counterpart). More robust evidence is warranted. Disentangling the role of epigenetics and its mediating genes may be clinically relevant. The in-depth clarification of the most relevant epigenetic signatures could allow risk assessment of severity and potential subsequent risk of mental disorders or health impairments, providing a prognostic tool. Moreover, and most importantly, the positive effect of an effective cognitive-behavioral treatment both on methylation levels and on reduction of anxiety^46^ could provide a tool for monitoring the effects of these treatments. Of utmost relevance here is that epigenetic modifications are reversible. One may even foresee a fruitful development of pharmacological and cognitive-behavioral interventions “personalized” to the epigenetic profile, to limit the negative consequences of sexual abuse. Finally, as stress-related psychopathology could cause accelerated cellular aging, further attention should be paid to epigenetics as a mediator of the detrimental effect of violence in the development of a wider range of long-term chronic diseases associated with aging (cancer, cardiovascular disease, and premature neurocognitive decline).^52^ More specifically, epigenetic clocks might be particularly relevant in this area, as they are powerful tools in the prediction of biological aging.

Physical abuse, especially sexual violence and domestic one, has been linked to psychiatric disorders, likely due to epigenetic perturbations. Indeed, HPA axis methylation might be compromised for stressful events such as violence.

### Childhood maltreatment and epigenetic impairment

Epigenetic modifications may also mediate, at least in part, the long-term effects of adverse childhood experiences on health outcomes later in life. The literature on childhood maltreatment is more extensive than the one on sexual abuse. A large body of evidence has already established a link between early life stress and a permanent impairment of either the serotonergic pathway or the HPA axis. However, only recently epigenetic mechanisms have been indicated as the possible bond between these two phenomena.

Based on the hypothesis that methylation of the 5HTT-promoter region could inhibit serotonergic responsiveness and serotonin transporter expression, favoring impulsive or aggressive behavior, Beach et al. investigated the possible link between this gene and antisocial behavior in 155 childhood-abused victims.^33^ Although the population was small, they were able to show a direct effect of childhood sexual abuse on 5HTT promoter methylation and adult antisocial behavior. They postulated that early life stressors may enhance vulnerability and facilitate long-term behavioral consequences.

In case of early stress exposure, the HPA axis may also be impaired. For instance, it is already well-known that female survivors of childhood maltreatment are more likely to have early menarche.^53^ It has also been postulated that the trajectory of cortisol levels is not linear: while cortisol might increase in the immediate aftermath of a stressful event, a potentially enhanced negative feedback mechanism could explain the subsequent condition of hypocortisolism later in life. Epigenetic mechanisms may be at play as a bridge between these events. One of the most studied genes in the HPA axis is the previously mentioned NR3C1, the glucocorticoid receptor implicated in the modulation of the HPA axis in response to stress. A systematic review on the role of this gene in child maltreatment confirmed a significant hypermethylation of NR3C1 gene in 8 of the 11 included studies,^25^ providing the correlate in peripheral blood of what was found in the brain tissue of suicide victims who had child abuse.^54^ The positive relationship seems also to have a dose-dependent pattern according to the intensity and frequency of the episode.^21^ In a population of psychiatric disorders (MDD, PTSD, borderline personality disorder), these authors showed that repetition of abuses correlated with the intensity of NR3C1 methylation status in blood samples collected. This aspect is clinically relevant because child abuses are seldom isolated events, whereas repeated exposures are usually the norm.^55^

In this context, the Black Women’s Health Study on the hypermethylation profile of NR3C1 in a cohort of 295 women reporting childhood victimization deserves utmost consideration.^23^ The study reported two intriguing findings as follows: increased methylation of NR3C1 promoter in those who were previously abused with a dose–response pattern for severity of abuse (methylation was two-percentage points higher in a most violent rather than lower intensity event), and ameliorative effect of emotional support on methylation magnitude. The study from Shields et al. was the first to correlate the methylation profile of this gene with childhood victimization, despite some limitations (*i.e.*, relatively small sample size, no difference between single and multiple episodes, potential recall bias).^23^ These results stand apparently in contrast with those reported 3 years later, in a cohort of 103 middle-aged women. In this setting, no association between NR3C1 methylation and early abuse was observed.^27^ However, caution should be taken when interpreting these specific results, as these two studies included different populations of abuse survivors; whereas in Shield et al., physical and sexual abuse was the prominent type of abuse, it was quite low represented in the Fiacco et al. cohort. Therefore, depending on the nature of violence, epigenetic modulation might be differently perturbated. Nevertheless, Fiacco et al. have also the merit to point out that gender could at least partly explain the different susceptibility to trauma-related impairment; they described a positive association between intensity of abuse and methylation status of the estrogen receptor (Erα) gene, postulating a role of estradiol in mediating the association between NR3C1 methylation and HPA-axis response.^27^ The dose and intensity pattern, the beneficial effect of support, and the gender-based susceptibility all together pave the way for a possible epigenetic-guided therapeutic approach.^56,57^

Another gene that has been studied is FKBP5, a strong stress-responsive protein that inhibits cortisol production with negative feedback on glucocorticoid receptor expression that might exert a role in various neuropsychiatric disorder risks.^58^ Based on a sample of 141 patients with psychiatric disorders, Misiak et al. described low methylation of FKBP5 gene in those who experienced child abuse.^28^ However, contrasting results emerged from a cohort of 4000 women where FKBP5 methylation was not significantly altered in patients with depression exposed to early victimization.^29^ Nevertheless, it should be reminded that Misiak et al. provided data on a cohort of psychiatric patients likely under therapy,^28^ while Konig et al. inquired only potential depressive symptoms in a general population.^29^ These contrasting aspects further highlight the urgency of homogeneous studies with similar time points, symptoms considered, and accurate confounder correction, to draw robust conclusions.

A recent article combining epigenetic clocks (Horvath, Hannum, PhenoAge, GrimAge) and data on cortisol secretion has drawn further attention to the nonlinear trajectory of the HPA function and the possibility of dynamic changes over time.^38^ In a prospective cohort of 132 women, the authors observed the variation in cortisol concentration and epigenetic aging over time (almost 40 years), providing inter- and intraindividual sampling data: a marked epigenetic aging acceleration was reported in those with the most pronounced deviations in HPA function following childhood victimization.^38^ Although this finding was observed in all four epigenetic age-acceleration clocks studied, some models (Horvath clock) were more sensitive to cortisol concentrations, as they included glucocorticoid-sensitive CpGs among the genes tested,^15^ while others (PhenoAge) were more effective in tracking time, as they were developed to register both chronological and biological aging.^59^ As postulated by the authors, epigenetic biomarkers of aging may provide the bridge between potentially reversible variation and stable psychophysical impairment in the victim, likely guiding targeted prevention.^38^

Indeed, postmaltreatment, HPA dysfunction may impair healthy biological aging, decades before the development of serious disorders.^35^ In this study in fact, early victimization and early menarche independently predicted a high risk of epigenetic age acceleration (based on the four epigenetic clocks aforementioned) in 183 premenopausal women. These results confirm the potential impact of HPA dysfunction on the timing of puberty and poor health later in life. Besides, the same group confirmed these preliminary findings in a larger cohort (*n* = 385 women), reporting an indirect effect of adverse childhood experiences on accelerated aging through menarche age. This body of evidence supports premature pubertal maturation as a compensatory mechanism to increase reproductive opportunities in a compromised longevity perspective, as indexed by biological markers.^37^

Despite this intriguing and promising evidence, there may be a question as to whether the epigenetic findings in the case of childhood maltreatment may reflect an adverse socioeconomic context rather than being the consequence of the violence itself. This concern was addressed by Lawn et al., who compared epigenetic age in a cohort of 1762 women, divided into two groups as follows: those from low socioeconomic backgrounds and survivors of childhood maltreatment. While no effect of socioeconomic deprivation on age acceleration was reported, sexual abuse was strongly associated with age acceleration by 3 years.^34^ In contrast, in a retrospective study based on 450 women, another epigenetic clock model (GrimAge clock), reported accelerated biological aging only in those who experienced poverty, hastening biological aging by 2 years, rather than abuse during childhood.^36^ Finally, in a large cohort of 2,232 twins assessed at 5, 7, 10, 12, and 18 years, a strong association between early victimization and epigenetic differences was not observed, either on EWAS or candidate gene screening (NR3C1, SLC6A4, FKBP5, AVP).^24^ However, the focus on twins, although mathematically attractive, could be viewed as an overmatching that could bury possible associations.

Altogether, these studies support a crucial role of epigenetics in mediating trauma-related effects but warn against further speculation, as it is likely that different epigenetic models might depict different traumatic aspects. Poverty and abuse might involve distinct loci. Traumatic events might also impair methylation only in a transient way, and resilience might have a protective effect on the epigenetic burden of those exposed to trauma. Pure psychological mechanisms could also be important confounders. Moreover, there is the need to address salient issues (which tissue is the most representative of the sample, which is the best assay, which is the best method of epigenetic analysis, what is the proper timing for assessment after the insult) to provide more robust evidence. Finally, because sensitive periods for epigenetic programming may exist,^60^ another key aspect could be the timing of the abuse. Sensitive periods represent developmental stages of increased plasticity, when experiences, including trauma, may have a more lasting impact. Indeed, studying the genes involved in opening, maintaining, and closing the plasticity phase of each specific biological pathway should be tackled before candidate gene analysis.^61^ The plasticity of child development is also an opportunity for overcoming early adversity, as much more eloquently recognized by the Institute of Medicine’s 2000 report, Neurons to Neighborhoods: “Virtually every aspect of early human development—from the brain’s developing circuitry to a child’s capacity for empathy—is influenced by the environment and experiences encountered in a cumulative manner, beginning in the prenatal period and extending throughout the early childhood years.”^62^

*Epigenetic changes of the HPA axis or of other elements as a consequence of childhood abuse may persist during life, leading to maladaptive adjustment. Epigenetic clocks could provide further insight into these mechanisms.*

### Generational inheritance of violence

Exposure to traumatic experiences at any stage of life (from childhood to adulthood) can not only affect the physical and psychological well-being of the victim but it can also have long-term effects on future generations. Traumatized women’s newborns are more likely to develop behavioral and clinical problems; in addition, children exposed to sexual abuse are likely to become abusive parents.^63^ Both considerations suggest the possible existence of an intergenerational impact of abuse. Epigenetic changes may play a role in this vicious intergenerational cycle of trauma and violence. If this is the case, there is an urgent need to identify which epigenetic aspects might be most informative about the potential for transgenerational inheritance. This could provide an opportunity for early stratification and intervention for high-risk offspring, allowing to target interventions.

NR3C1 was the focus of a project that aimed to investigate whether adverse pregnancy trauma could have a long-lasting effect on DNA methylation in the offspring. Looking at 24 mother–child dyads, Radtke et al. found a positive association between offspring NR3C1 promoter methylation status and maternal exposure to IPV during pregnancy, while it was not observed when the event occurred before or after pregnancy.^22^ In Brazil, a group of researchers looked at the extent to which epigenetic mutations might be persistent through generations, considering the grandmother–mother–newborn triad. In a population of 375 individuals, a decrease in the methylation of BDNF and CLPX was registered both in the traumatized mother and in the child, but not in the grandmother generation.^19^ Even if these results might suggest that epigenetic inheritance is a one-generation process, more robust evidence is needed.

The same group questioned whether epigenetics might be involved in contrasting the psychological burden of violence: children living in a Brazilian high violence community appeared to be more resilient to psychiatric consequences of prenatal IPV. In this setting, they observed that in a cohort of 122 children prenatally exposed to violence, NR3C1 and FKBP5 were most methylated, providing an enhanced ability to terminate hormonal stress responses. Indeed, this observation suggests that prenatal psychiatric risk factors may have different, and perhaps even opposite, consequences depending on the population and timing examined.^30^ This aspect introduces the potential role of epigenetic modification also in shaping resilience patterns in violence-survivors, partly explaining why not all victims develop psychiatric disorders. Interestingly, in a cohort of 114 pregnant women with a history of child maltreatment, Grasso et al. found that the severity of symptoms differed according to the genotype of FKBP5. Infants with the CC genotype were more protected when exposed to perinatal stressors, highlighting the complexity of the interplay between genetics, epigenetics, and exposure.^31^

Another possible way of biological embedding of traumatic experience is a general process of premature aging. In Congo, six different epigenetic clocks were applied to 151 mother–baby pairs exposed or not to 4 stressors (general, sexual, war trauma, and chronic).^39^ They found that in case of sexual trauma, epigenetic age acceleration was registered in mothers, whereas many differentially methylated positions (DMPs) were identified in both mother and newborn, confirming that not only the direct victim but also the indirect recipient received an epigenetic impact.^39^ To note, MUC4-DMP, which is linked, for example, to endometriosis chronic pain frequency,^64^ was the most frequent DMP in women who reported sexual abuse, but it was not impaired in the offspring.

The term “transmission” of trauma has already been used in the past to define the possible influence of parental experiences on the feelings and psychological well-being of newborns.^65,66^ The epigenetic data on the possibility of transgenerational inheritance make this aspect not only relevant from a theoretical point of view but may also lead to the development of new and effective interventions to help minimize the consequences of trauma in survivors and their offspring.

*Life-experience, especially traumatic one such as abuse, might interfere not only with victim’s health but also favor epigenetic drift that could be inherited to newborns. Indeed, it could also cause accelerated aging process in the newborns.*

## Discussion and Future Perspective

A new emerging line of research is investigating whether environmental stressors, such as abuse, could affect health through epigenetic effects. In other words, it has been suggested that risk of permanent disease, as a result of abuse, could be partly attributed to epigenetic changes. In this review, we present the initial evidence on this topic with respect to three possible victims as follows: women, children, and future newborns (Fig. 1).

Based on available evidence, two main additional reflections on epigenetic signatures and violence can be drawn.

First, environmental stressors are responsible for the disorders, but the nature of the effects might be influenced by susceptibility genes and psychological background, as well as described by the “gene-environment theory.” This could explain the heterogeneity in how subjects may respond to trauma. In other words, certain victims are highly vulnerable, whereas others may develop resilience. Epigenetic modifications could be a mediator of the effect of traumatic episodes. From this point of view, epigenetic traits might allow screening systems to recognize those victims who are at higher risk and who require tighter control. However, the precise role of epigenetics in the consequences of violence is multifaceted and not yet definitely clarified.

Second, epigenetics is a dynamic change of genome expression, under the influence of the environment, but also a hereditary trait. This aspect could have two different implications: first, it could justify why the effects are not only limited to the victim but also transmitted to newborns; second, it provides the reason why this field is not only interesting from a theoretical standpoint, but also from a pragmatic one. The focus on modifiable factors can be used as a starting point for preventive and curative interventions, allowing to potentially limit the negative effects of trauma.

To unravel the complexity of the issue, a better understanding of the compound pathways that underlie these relations is crucial. Here by, we sum up the most relevant questions to address, based on the review provided:

I. Genome analysis: Which are the genes more susceptible to violence? Is it possible that different forms of violence could interact with different genes? Could epigenetic clocks provide the same or different information rather than single-gene epimutations?
II. Pathogenic analysis: Which is the best timing to study epigenetic effects? How long do epimutations last? Do sensitivity period and genes have a role and in case, which one?
III. Methodology considerations: Which is the best technique: single-candidate gene versus whole-epigenome sequencing? What are the limitations of cross-sectional studies compared with longitudinal observational ones?
IV. Long-term perspective: What are the relative effects of psychological background and epigenetic modifications in the determinisms on long-term detrimental effects on health?
V. Therapeutic strategy: Could health/psychotherapeutic approaches potentially ameliorate epigenetic modifications? If so, which approaches are most effective, and to what extent? Which are the ethical implications raised by this new approach? And how could epigenetic targeted therapies be developed, given the complex and multiple consequences of violence?

## Conclusion

Every form of physical or psychological violence is a violation of human rights. Preventing this type of trauma and reducing its possible harmful consequences are therefore a priority for global health policies. The “elimination of all forms of violence against women” has been included as a goal in the 2030 Agenda for Sustainable Development.^67^ Every effort should be made to counteract this epidemic, to prevent recurrence, and to persecute the perpetrators. However, other actions are also warranted. The awareness of this violent pandemic should be translated into immediate efforts to understand and minimize the consequences of traumatic experiences suffered by victims, whether adult or child. In fact, it has already been proved that any form of abuse has an impact on psychological and physical well-being also later in life and can affect longevity and health. The possibility that the victim may somehow also pass this “traumatized trait” to the offspring amplifies the relevance of the issue. From this perspective, epigenetics could play a crucial role in the natural history of violence. Studies should first try to answer preliminary questions, as already highlighted. Only once these premises are clarified, it will be possible to get “under the skin” of the violence world. Well-designed epidemiological research could shed light on the specific combinations of negative outcomes that are most observed and may provide tools that can better tailor interventions to prevent or reduce the magnitude of such problems. More in general, to really improve our knowledge and understanding of the pathways to negative outcomes among victims of gender-based or childhood violence, there is the need for an integrated approach that incorporates psychological, epidemiological, and biological perspectives into more comprehensive translational models.

## Abbreviations Used

5HTT: Serotonin transporter
ADCYAP1: adenylate cyclase-activating polypeptide 1
BDNF: Brain-derived neurotrophic factor
BRSK2: brain-specific serine /threonine-protein kinase 2
CLPX: caseinolytic mitochondrial matrix peptidase chaperone subunit
DMP: differentially methylated positions
DRD2: D2 dopamine receptor
ERα: Estrogen receptor
EWAS: epigenome-wide association study
FKBP5: FK binding protein 5
HPA: hypothalamuspituitary adrenal
IGF2: Insulin-like growth factor 2
IPV: intimate partner violence
MAO: monoamine oxidase A
MDD: major depressive disorder
miRNA: microRNA
NR3C1: nuclear receptor subfamily 3, group C, member 1
PTSD: post traumatic stress disorder
